# Machine learning approaches to anxiety detection: trends, model evaluation, and future directions

**DOI:** 10.3389/frai.2025.1630047

**Published:** 2025-10-21

**Authors:** Meruyert Taskynbayeva, Alina Gutoreva

**Affiliations:** Kazakh-British Technical University, Almaty, Kazakhstan

**Keywords:** anxiety prediction, machine learning, mental health diagnostics, anxiety symptoms, mental health

## Abstract

**Background:**

Anxiety is a pervasive mental health disorder with severe implications for individual wellbeing and societal productivity. The contemporary rise of anxiety, particularly among youth in digitally-saturated environments, underscores a critical need for advanced predictive tools to facilitate early intervention and mitigation. While machine learning (ML) holds significant promise in this domain, a comprehensive synthesis of its application in anxiety prediction, along with a critical evaluation of methodological trends and gaps, is only emerging in the literature. The main idea of the current systematic review is to bridge the understanding of current ML applications in mental health with the critical needs for enhanced diagnostic precision, personalized interventions and prevention.

**Objectives:**

This systematic review aims to systematically synthesize research on ML approaches to predicting anxiety, critically evaluating the algorithms, features, and validation techniques employed across studies. The objective is to identify prevailing ML techniques, assess their performance, and highlight crucial methodological trends, existing gaps, and their implications for effective early intervention and real-world deployment.

**Eligibility criteria:**

Studies included had to apply machine learning techniques to predict anxiety or its severity using either clinical or behavioral datasets. Exclusion criteria included non-English language papers, reviews, older or previously reviewed publications, and those not specifically targeting anxiety. We focus on questionnaire research, but also discuss multimodal fusion techniques.

**Information sources:**

We searched the Scopus database and Google Scholar for articles published between 2018 and 2025 using combinations of keywords including “anxiety prediction,” “machine learning,” and “mental health.” The last search was conducted in July 2025.

**Risk of bias:**

Studies were screened in two phases: (1) by verifying the presence of relevant keywords in the main body, and (2) by reviewing title, introduction, and conclusion to ensure alignment with anxiety prediction via ML. Studies relying solely on self-reported metrics or with unclear algorithmic transparency were noted for potential bias.

**Results:**

A total of 19 studies were included, encompassing 44, 608 participants. GAD-7 and DASS-21 were the most commonly used diagnostic instruments. ML techniques such as Random Forest and Gradient Boosting achieved the highest predictive accuracy, with some studies reporting up to 98% accuracy. Metrics like F1-score, AUC, and specificity were commonly reported.

**Limitations of evidence:**

Existing studies display a range of methodological and conceptual limitations that constrain their generalizability and clinical utility. The review identified significant methodological limitations hindering generalizability and clinical utility, including reliance on small, homogeneous samples, which raises concerns about overfitting and population bias. Furthermore, common issues include a lack of external validation, inconsistent evaluation metrics, and the “black-box” nature of many ML algorithms, which impedes clinical trust and adoption.

**Interpretation:**

The findings support the effectiveness of machine learning for anxiety detection and prediction, particularly in early intervention contexts. The integration of explainable ML and diverse, clinically validated data is necessary for real-world deployment. The existing body of research also shows a notable scarcity in studies predicting anxiety before symptom manifestation. These insights emphasize the critical need for integrating explainable ML (XAI) and utilizing diverse, clinically validated datasets to enable real-world deployment and proactive mental health support.

## Introduction

“*The ‘anxious generation' faces new pressures unlike any before, where virtual social lives can provoke intense stress and worry about acceptance and self-worth.”* — Haidt, 2024, p. 58

Anxiety is considered a future-oriented, long-acting response broadly focused on a diffuse threat, whereas fear is an appropriate, present-oriented, and short-lived response to a clearly identifiable and specific threat (American Psychological Association, 2018). Dedicating more resources and attention to the topic of anxiety can ultimately assist individuals in leading happier, healthier lives. Findings of research suggest that anxiety significantly influences individuals' self-perceived health and overall wellbeing, much like depression (Hanna and Strober, 2020). This is a significant concern because anxiety can ultimately lead to suicide. To emphasize this point, Nepon et al. (2010) concluded that more than 70% of respondents who had attempted suicide at some point in their lives also had experienced an anxiety disorder.

Recent cultural analyses, such as Haidt's *The Anxious Generation* (2024) highlight a growing mental health crisis among youth, particularly anxiety-related disorders, in relation to digital environments and social media exposure. This sociocultural backdrop further motivates the application of machine learning techniques to better understand, predict, and mitigate anxiety in younger populations. In the following section, we examine machine learning methodologies used in mental health research, with particular attention to how they might offer insights into the behavioral patterns, risk factors, and intervention pathways for anxiety disorders in digitally immersed generations.

The symptoms of anxiety encompass a range of experiences that can significantly impact an individual's wellbeing. These include excessive worry, which often manifests as persistent thoughts about potential future events (Fell et al., 2023; Stapinski et al., 2010; Wells, 1995; Mathews, 1990; Stein and Sareen, 2015; Hoge et al., 2012). Physically, anxiety may manifest in physiological responses such as an increased heart rate, sweating, muscle tension, and headache (Stein and Sareen, 2015; Crawley et al., 2014; Hoge et al., 2012; Pary et al., 2003). Additionally, individuals experiencing anxiety may find themselves withdrawing from social interactions, leading to a sense of isolation (Teo et al., 2013). Sleep disturbances, particularly insomnia, are common manifestations of anxiety, as the mind races with persistent thoughts, making it challenging to relax (Crawley et al., 2014; Stein and Sareen, 2015; Hoge et al., 2012; Pary et al., 2003). Moreover, symptoms include difficulty concentrating and irritability (Pary et al., 2003; Vidal-Ribas et al., 2016; Stringaris, 2011).

Detection of anxiety refers to the process of identifying or recognizing the presence, symptoms, or indicators of anxiety disorders or heightened anxiety levels in individuals. Prediction of anxiety involves forecasting or estimating the likelihood, severity, or future progression of anxiety-related conditions in individuals. Detecting anxiety involves recognizing current symptoms or indicators, which then inform predictive models for estimating future anxiety levels or risk. Anxiety can have various symptoms. Physically, it might mean a racing heart, tense muscles, or trouble breathing. Mentally, it can bring excessive worry, difficulty concentrating, or racing thoughts. Emotionally, it might result in irritability, fear, or sudden panic. Behaviorally, it could lead to avoiding certain situations or experiencing trouble sleeping. Recognizing these symptoms helps build models that estimate future anxiety levels or risks in individuals.

One way of mitigating the impact of anxiety could be early detection. Research by Hanna and Strober (2020) indicates that early identification of anxiety reduces its effects. Notably, machine learning algorithms have proven their power in diagnosing anxiety. For example, a study by Priya et al. (2020) successfully predicted anxiety, defined the Random Forest algorithm as the best, and has been foundational work for subsequent research in the past 3 years. In one such recent study, the Marine Predators Algorithm and kNN demonstrated a high accuracy of 98.11% in detecting anxiety and depression in pregnant women (Ögür et al., 2023). Moreover, in a separate study involving a sample of 3,984 students aged 10–15 years in fifth to ninth grades, researchers made remarkable progress in predicting anxiety, achieving an impressive accuracy rate of 92.4% (Qasrawi et al., 2022). In a different study conducted by Sajja (2021), the anxiety levels of 600 university students were predicted using machine learning techniques, reaching an accuracy rate of 94%. Additionally, Ratul et al. (2023) concentrated on predicting psychological and social stress levels. In this research, machine learning models demonstrated remarkable accuracy, achieving 80.5%. These results emphasize the efficiency of machine learning in diagnosing and categorizing anxiety, highlighting its fundamental role in addressing mental health issues.

Artificial intelligence (AI) and machine learning (ML) are integrated now in diagnostic assistance. By integrating behavioral and physiological data from digital methods, the approach enables passive, real-time detection of anxiety risk. The methodological innovation is in the use of interpretable temporal models, grounded in computational clinical psychology and psychiatry, to capture symptom trajectories and environmental triggers. This work offers generalizable lessons for embedding ML within educational informatics to enable proactive, data-driven mental health support. ML has progressively transformed the understanding and treatment of mental health disorders, tracing its roots back to early computational models of human communication and decision-making. One of the pioneering efforts in this domain was Weizenbaum (1966) development of ELIZA, an early natural language processing program simulating therapeutic conversations, which laid foundational groundwork for human-computer interaction in mental health contexts. Subsequent advances in probabilistic reasoning and Bayesian models, exemplified by Charniak and Goldman (1993), further contributed to the computational modeling of complex psychiatric diagnosis processes. More recently, machine learning techniques have been extensively applied in clinical psychology and psychiatry to enhance diagnostic accuracy and predict treatment outcomes (Dwyer et al., 2018). The advent of digital phenotyping, as articulated by Insel (2017), has introduced novel methods for behavioral data collection via ubiquitous devices, enabling a new science of mental health that leverages continuous, real-world monitoring. Complementing these approaches, natural language processing applied to non-clinical texts offers promising avenues for detecting mental health signals outside traditional clinical settings, broadening the scope of computational psychiatry (Calvo et al., 2017). Together, these advances highlight a multidisciplinary convergence that is reshaping both the theoretical understanding and practical management of mental health conditions through AI.

The rapid development of machine learning (ML) has catalyzed significant advances in mental health diagnostics, particularly through secondary studies that synthesize evidence across empirical models. Systematic reviews and narrative surveys have become essential in revealing methodological trends, identifying bias sources, and guiding future designs in anxiety detection. Among the most influential recent studies are a systematic review targeting student populations (Schaab et al., 2024), a biosignal-centered survey of detection systems (Ancillon et al., 2022), and a broad scoping review on mental disorders and stress-related predictions (Razavi et al., 2023). Also, Schaab et al. (2024) examined 48 studies assessing ML-based detection of anxiety, depression, and stress in undergraduate students. While most models showed internal accuracy exceeding 70%, anxiety-specific performance varied from 53.7 to 98%. A key limitation noted was the near-total absence of external validation—only one study employed it—rendering most findings potentially overfit and context-specific. The authors applied the GRADE framework and rated the overall certainty of evidence as “very low,” emphasizing the gap between promising technical performance and clinical applicability.

Additional reviews provide further insight into ML's application across diverse populations and data sources. Pintelas et al. (2018) conducted a comparative review focused specifically on anxiety disorders, revealing that ML methods show substantial potential in disorder prediction, although accuracy varied by anxiety subtype and algorithm used. They reviewed 16 studies from 2010 to 2017. Similarly, Ahmed et al. (2022) reviewed 54 studies, from 2010 to 2021, leveraging social media data to detect anxiety and depression, particularly during the COVID-19 pandemic. Their findings highlighted how users' online behaviors and language patterns—collected from platforms such as Twitter, Facebook, Reddit, and Weibo—can inform predictive modeling. This approach holds promise for augmenting traditional screening tools, especially in contexts with limited access to mental health services.

Further expanding the landscape, Abd-alrazaq et al. (2023) conducted a systematic review and meta-analysis on wearable AI technologies for anxiety detection. Synthesizing results from 21 studies, they reported a pooled mean accuracy of 0.82, sensitivity of 0.79, and specificity of 0.92. While the performance was not significantly moderated by device type, algorithm, or validation method, the review concluded that wearable AI is not yet suitable for clinical deployment and should be used to complement—rather than replace—traditional assessments. Notably, the authors called for more refined tools capable of real-time anxiety detection and differentiation between anxiety subtypes.

A complementary survey by Ancillon et al. (2022) explored anxiety detection via physiological biosignals, including EEG, ECG, electrodermal activity (EDA), and respiration. They reviewed studies from 2012 to 2022, with sample sizes ranging from 10 to 102 and reported accuracies between 55 and 98%. The highest-performing models typically integrated multiple signals—especially EDA and heart rate—suggesting that multimodal approaches outperform univariate models. Despite this, limitations persisted: small datasets, lack of benchmarking, and inconsistent preprocessing protocols hindered generalization. Traditional ML classifiers such as random forests and SVMs remained dominant, while neural networks were increasingly adopted for automated feature learning. Expanding the scope, Razavi et al. (2023) conducted a scoping review of 98 studies focused on stress and related disorders, reinforcing earlier observations. The authors noted strong methodological convergence—reliance on supervised learning, limited personalization, and rarely interpretable models. Issues such as scarce real-world deployment, missing transparency metrics, and poor adaptation to contextual variables remain common.

These studies converge on four recurring themes. First, most models exhibit high internal accuracy but suffer from limited external validation, undermining their readiness for deployment (Schaab et al., 2024; Ancillon et al., 2022; Abd-alrazaq et al., 2023). Second, biosignal fusion—especially EEG, ECG, and EDA—significantly improves predictive performance, though such methods remain largely confined to controlled lab settings (Ancillon et al., 2022; Abd-alrazaq et al., 2023). Third, the widespread use of tree-based and neural architectures reveals a methodological plateau, while interpretability, fairness, and contextual modeling are underprioritized (Razavi et al., 2023; Pintelas et al., 2018). Fourth, the lack of standardized feature engineering, preprocessing pipelines, and benchmarking frameworks continues to fragment the field (Razavi et al., 2023; Ahmed et al., 2022).

These findings inform our conceptual pipeline design for anxiety prediction rooted in behavioral science. Specifically, they stress the need for external validation protocols, multimodal data integration, and transparent model architectures. Embedding psychological theory and intersectional sensitivity at each pipeline stage may help resolve persistent challenges and align AI systems more closely with human experience.

We invite the reader to think about trying to predict a storm: current ML models are excellent at recognizing the signs of an approaching storm (detecting anxiety symptoms) and even forecasting its intensity once it's visible (predicting severity). However, this review highlights that we're still building the tools to reliably predict a storm before the clouds even gather on the horizon, and we need to ensure our weather stations (data sources) are diverse and our forecasting models (algorithms) are transparent enough for meteorologists (clinicians) to truly trust and act upon their predictions.

We structured our inquiry around one primary research question, supported by three secondary questions that guide and deepen our discussion. These research questions are as follows:

1. (1) To what extent has machine learning (ML) been used to predict anxiety, and how comprehensively has this area been explored in the literature?
And then the secondary questions:
2. (1.1) What machine learning methods have been applied to anxiety prediction, and which have demonstrated superior performance?
3. (1.2) Which feature selection algorithms are most commonly utilized in building predictive models for anxiety?
4. (1.3) What evaluation metrics are used to assess the accuracy and effectiveness of ML models in anxiety prediction?

The increasing number of individuals having anxiety creates the need to improve the prevention and treatment strategies. As discussed above, Machine learning techniques are showing good results in predicting the disorder, which could prevent the deterioration of the symptoms. However, there is a lack of investigations of studies focusing on predicting anxiety with Machine learning and it is a fairly new area of research.

This systematic review synthesizes existing research on machine learning (ML) approaches for anxiety prediction by critically examining the algorithms, feature sets, and validation techniques employed across studies. The primary objective is to identify the most commonly used ML models, evaluate their predictive performance, and extract key methodological trends. In doing so, the review also highlights significant limitations, research gaps, and implications for early detection, clinical implementation, and real-world deployment of ML-based anxiety prediction systems. Ultimately, this paper aims to provide a comprehensive understanding of the current landscape of ML applications in anxiety prediction–identifying successful strategies, recurring challenges, and opportunities for methodological advancement. The study is intended to serve as a valuable resource for mental health professionals, machine learning practitioners, software developers, and researchers in psychology, psychiatry, and computational healthcare.

## Methodology

We utilized the Scopus database and Google Scholar for our paper analysis. Scopus, an abstract and indexing database, contains peer-reviewed literature with full-text links. It was named after the Hammerkop bird, known for its exceptional navigation abilities. The Scopus records span back to 1,788 and comprise over 44,000 titles from around 7,000 publishers. Among these titles, nearly 35,000 are peer-reviewed journals in various subject areas. Scopus encompasses a range of formats, including books, journals, and conference papers, across fields such as science, technology, medicine, social sciences, and arts and humanities.

A search query looks as follows: (“anxiety predict*” OR “predict* anxiety” OR “predict* depression” OR “predict* distress”) AND (“machine learning” OR “artificial intelligence” OR “data mining” OR “predictive modeling”). It produced 255 results. Depression and distress are other mental health disorders that are often studied in conjunction with anxiety. It is important to note that by excluding the terms “depression” and “distress” from the query and searching for “(“anxiety predict*” OR “predict* anxiety”) AND (“machine learning” OR “artificial intelligence” OR “data mining” OR “predictive modeling”),” we obtained a reduced set of only 55 results. This represents approximately 20% of the total results. This suggests that comprehensive research on machine learning predicting anxiety is still in its infancy.

**Figure 1:**
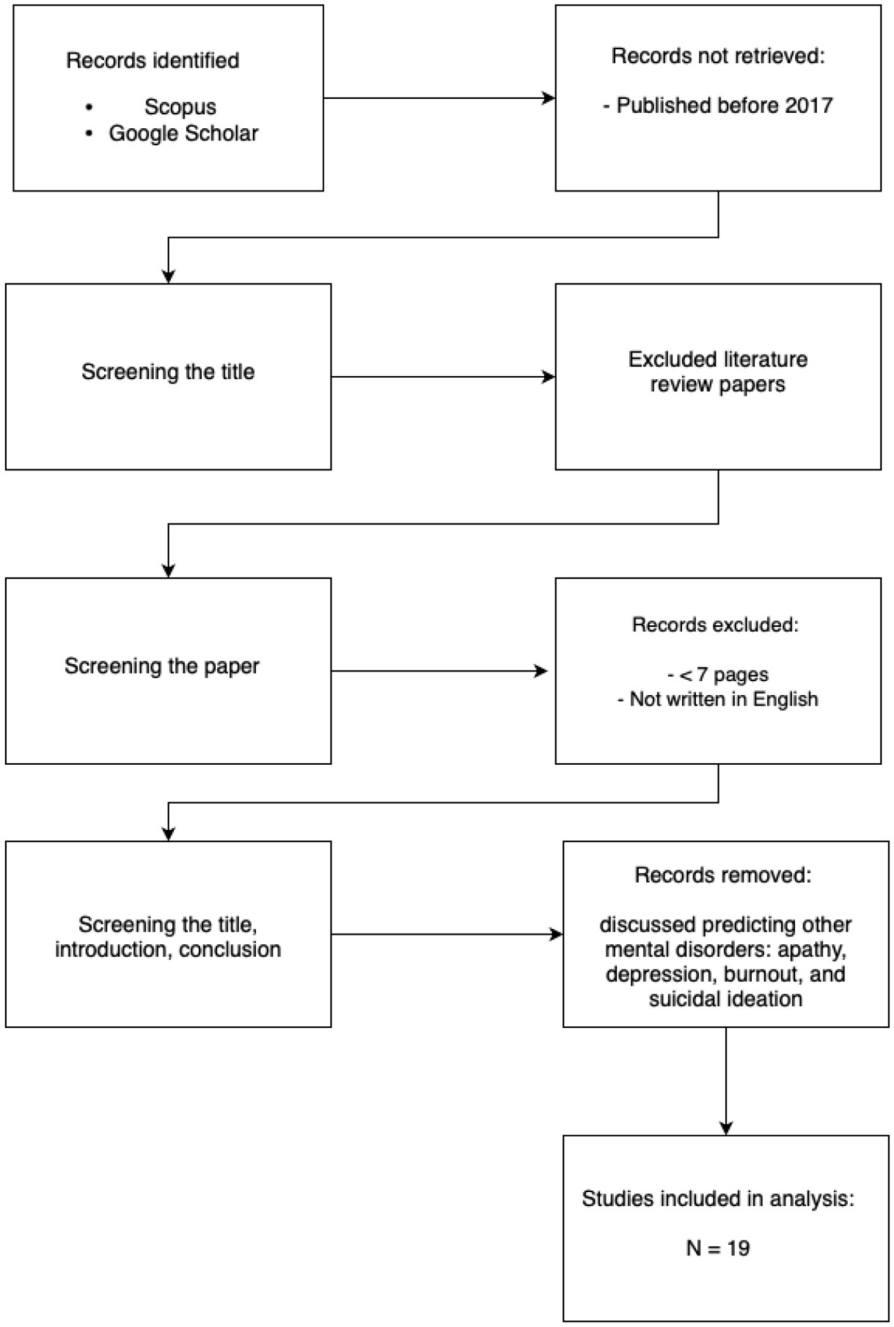
PRISMA flowchart describing the selection process of papers analyzed in the current review. It is noteworthy that while recent advancements demonstrate the growing role of AI in mental health, at least thirteen foundational and review papers explicitly comment on the persistent limitations in our understanding of core psychiatric disorders, particularly anxiety and depression. These works often highlight the relatively low volume of targeted research in this space compared to other medical domains, signaling both a challenge and an opportunity for more rigorous, interdisciplinary investigation.

Google Scholar is a freely available service that allows to search for scholarly literature - scientific papers, articles and books, which is an academic version of Google. It was built 20 years ago, in 2004 by Anurag Acharya and Alex Verstak, the same year as the Scopus database. We used the search query “machine learning” AND “anxiety prediction.” The results were filtered to include only recent years (2018–2025). The papers retrieved went through a two-step process to determine whether they should be excluded based on specific exclusion criteria. In the first step, we checked whether the keywords “Anxiety” and “Machine learning” were used and excluded publications where they were used only in the bibliography. Furthermore, papers that were too short (less than 7 pages) and not written in English were excluded. In the second step, we read the title, introduction, and conclusion of each paper. The papers that do not research the topic of predicting anxiety with ML were excluded. Among those were publications that discussed predicting other types of mental disorders such as apathy, depression, burnout, and suicidal ideation along with diseases like cancer, dementia, heart disease, etc. Additionally, we excluded literature review papers (see Figure 1). Moreover, the publications that were old and have already been analyzed in previous literature reviews were also excluded, so we focused on new research.

Despite notable progress in the application of AI to mental health, a critical review of the literature reveals that at least thirteen key a lack of the use of these technologies in foundational understanding of psychiatric disorders such as anxiety. These papers frequently point to a disproportionately lower research focus in computational psychiatry compared to other medical domains. This imbalance highlights a need for more robust, theory-driven research that integrates clinical knowledge with advanced AI methodologies, thereby strengthening the translational impact of these technologies.

In the following section, we explore machine learning methods that have been applied to mental health research, with a particular focus on their potential to improve diagnostic precision, enable personalized interventions, and uncover latent patterns in behavioral and clinical data.

### Overview of machine learning anxiety prediction techniques

A variety of Machine Learning (ML) algorithms have been employed to predict anxiety and related mental health outcomes, utilizing diverse datasets and methodological approaches. Machine learning broadly encompasses three main types: Supervised, Unsupervised, and Reinforcement Learning. Among these, Supervised machine learning is predominantly used for making predictions and relies on structured, labeled training data. The studies reviewed in the sources demonstrate a strong methodological convergence, indicating a reliance on supervised learning for anxiety prediction. This approach is well-explored for classification tasks in machine learning. In contrast, Unsupervised machine learning is generally employed to understand relationships within datasets, while Reinforcement Learning is used to enable models to choose actions within an environment to maximize rewards in particular states. These distinctions are important for understanding the different applications of machine learning approaches in predicting anxiety.

Below, we will examine several different types of ML models and comprehensively explore their respective strengths and limitations. This includes both classical algorithms—such as decision trees, support vector machines, and logistic regression—and more complex architectures like deep neural networks and fuzzy systems. Each class of model offers distinct trade-offs: while some excel in interpretability and simplicity, others prioritize predictive performance at the cost of transparency. Understanding these differences is essential when selecting models for sensitive domains such as mental health, where both accuracy and explainability are crucial.

**Support Vector Machines (SVM)** are particularly valuable for anxiety prediction due to their ability to effectively handle high-dimensional, low-sample-size data—a frequent challenge in clinical psychological datasets. SVMs excel in binary classification tasks such as distinguishing between anxious and non-anxious individuals. Kernel functions such as the radial basis function (RBF) allow SVMs to model complex, nonlinear relationships between behavioral, physiological, or multimodal input features and anxiety states (Cortes and Vapnik, 1995; Noble, 2006). This is critical when working with heterogeneous features (e.g., EEG, facial expressions, self-reports) that often exhibit nonlinear correlations with anxiety. Prior studies have demonstrated SVMs' superior performance in clinical prediction tasks, including anxiety disorder classification, compared to other machine learning models (Gavrilescu and Vizireanu, 2019).

**Figure 2:**
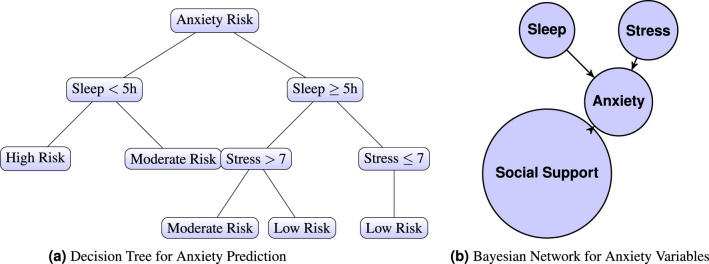
**(a)** Decision Tree and **(b)** Bayesian Network for Anxiety Prediction. Sleep and stress are widely recognized as critical factors influencing the risk of anxiety. Increased sleep deprivation and heightened stress levels both elevate the likelihood of anxiety symptoms manifesting **(b)**. A Bayesian Network for Anxiety Variables illustrates the interrelationships between key factors: “Sleep,” “Stress,” “Social Support,” and “Anxiety.” In this network, “Sleep” and “Stress” are variables that potentially influence the development of anxiety, while “Social Support” acts as a mitigating factor. The arrows in the network represent causal relationships. Specifically, both “Sleep” and “Stress” are shown to have a direct influence on “Anxiety,” as indicated by arrows pointing from insufficient sleep and heightened stress to anxiety. Notably, the arrow pointing from “Anxiety” to “Social Support” is reversed, reflecting literature that suggests higher anxiety can negatively impact social engagement, thereby reducing social support.

**Decision Trees**, with their transparent and hierarchical decision logic, are frequently employed in mental health research, particularly when model interpretability is critical. This is especially important in clinical and psychological settings, where explainable outcomes can inform therapeutic decision-making and increase clinician trust (Lundberg and Lee, 2017). Decision Trees enable the tracing of anxiety-related decision paths, allowing researchers and practitioners to identify threshold values in stress, sleep quality, or physiological signals that contribute to high-risk classifications (Figure 2a). Their capacity to visualize branching logic and feature importance makes them well-suited for analyzing multi-factorial mental health data in a way that is both human-interpretable and diagnostically useful (Pratt et al., 2016; Wu et al., 2020).

Here it also important to mention other **Tree-Based Models and Boosting Techniques**. Benito et al. (2024). used XGBoost for classifying anxiety severity based on self-report and environmental variables. SHAP values were utilized to enhance interpretability. Qasrawi et al. (2022) and Bhatnagar et al. (2023) applied Gradient Boosting (GB) and Random Forest (RF) models. GB minimized additive loss functions, while RF relied on majority voting. Sau and Bhakta (2017) tested multiple classifiers, including Random Forest and Naive Bayes, with RF outputs derived from aggregated tree votes. Li et al. (2024) employed a Random Forest with Under-Sampling (RF-US) algorithm, which predicts outcomes based on an ensemble of decision trees, each voting for the most probable class. Under-sampling was specifically applied to balance class distribution by randomly reducing the majority class samples. Hyperparameters were optimized through Bayesian optimization minimizing the out-of-bag error. Random Forest constructs multiple decision trees on subsets of data and aggregates predictions through majority voting.

**Bayesian Networks**, are especially useful in modeling probabilistic relationships among psychosocial variables such as stress, sleep, and social support (see Figure 2b). Their strength lies in explicitly representing conditional dependencies, enabling scenario testing (e.g., how does improved sleep modify anxiety risk under high stress?), as shown in Equation 1.


(1) $$\begin{array}{l} P\left(X_{1},\ldots ,X_{n}\right)=\overset{n}{\underset{i=1}{\prod }}P\left(X_{i}|\text{pa}\left(X_{i}\right)\right) \end{array}$$

where pa(*X*_*i*_) denotes the set of parent nodes of *X*_*i*_ in the Bayesian Network graph.

In regards to anxiety, **Artificial Neural Networks (ANNs)** shine. They model complex interactions and learning hidden representations from behavioral, physiological, and text-based features that are so important to combine for accurate diagnosis, prevention of anxiety. ANNs are highly adaptable and capable of incorporating multimodal data streams (e.g., GSR, speech, smartphone usage) for dynamic anxiety prediction. The foundational transformation is shown in Equation 2, where feature weights and bias are optimized through backpropagation to minimize prediction error. ANNs have become a cornerstone in anxiety prediction research due to their ability to model nonlinear, high-dimensional relationships across diverse input features. Studies such as Priya et al. (2020) and Schaab et al. (2024) have demonstrated the effective use of ANNs for classifying anxiety severity based on behavioral, self-reported, and physiological indicators. ANNs are particularly valuable in settings where patterns of anxiety symptoms are subtle, temporally variable, and influenced by complex contextual cues. A simple ANN is given in Equation 2:


(2) $$\begin{array}{l} z=f\left(\overset{n}{\underset{i=1}{\sum }}\left(w_{i}\cdot x_{i}\right)+b\right), \end{array}$$

where *f* is an activation function such as the sigmoid, tanh, or ReLU. Here, *z* represents the neuron's output, *x*_*i*_ are input features such as sleep quality, stress level, or heart rate variability, *w*_*i*_ are learned weights indicating the importance of each input, and *b* is a bias term allowing the network to fit data more flexibly. During training, the model updates *w*_*i*_ and *b* to minimize prediction error, typically via backpropagation and gradient descent. ANNs have proven particularly useful in fusing multiple modalities. For instance, Ancillon et al. (2022) reported that neural networks achieved high accuracy in anxiety detection when combining biosignals (e.g., GSR, ECG) with speech-derived features, even outperforming traditional classifiers in multimodal settings. Razavi et al. (2023) further emphasized their scalability in analyzing large time-series datasets from wearable sensors, enabling passive and continuous monitoring of anxiety symptoms.

Despite their strengths, a common criticism of ANNs is their opacity–the so-called “black-box” problem. This poses challenges in clinical applications where interpretability is essential. However, recent advances in Explainable AI (XAI) are beginning to mitigate this concern. Notably, SHapley Additive exPlanations (SHAP) and Layer-wise Relevance Propagation (LRP) allow for attributing a model's output to specific input features, enabling practitioners to understand whether anxiety predictions are driven by elevated heart rate, disrupted sleep, or sudden behavioral shifts (Lundberg and Lee, 2017). These methods improve trust and transparency in neural models applied to mental health. ANNs are particularly well-suited for anxiety prediction due to their ability to:

- capture complex, nonlinear dependencies among psychophysiological and behavioral features,
- scale across datasets with high input dimensionality,
- integrate multimodal signals effectively, and
- adapt to real-time streaming data for continuous monitoring, so important for digitalised applications.

These capabilities make ANNs a compelling tool in both research and clinical applications for mental health, especially when paired with interpretability tools to foster trust and transparency.

**Figure 3:**
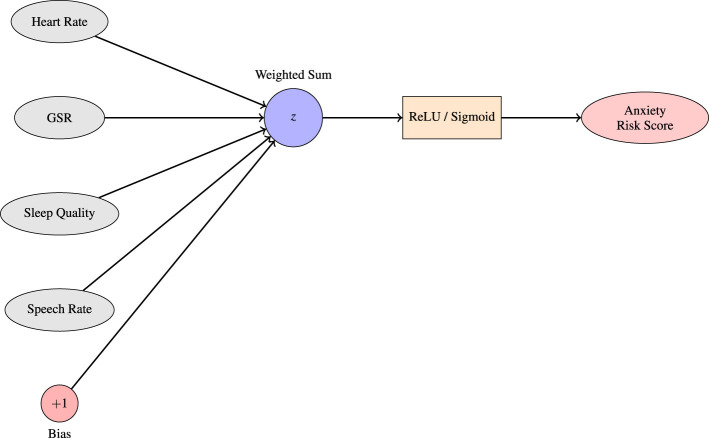
A single-layer perceptron serves as a foundational model for predicting anxiety levels based on physiological inputs. In this setup, each input node represents a specific biomarker associated with anxiety, such as heart rate, galvanic skin response (GSR), sleep quality, and speech rate. These inputs, each assigned a corresponding weight reflecting their learned contribution to the prediction task. The bias term allows the decision boundary to shift. This linear combination *z* is then passed through a nonlinear activation function, such as the sigmoid or ReLU and produce a final anxiety risk score. The resulting output reflects the likelihood or intensity of anxiety in the subject based on the input features. This basic structure forms the computational unit for more complex neural architectures employed in affective computing and mental health prediction systems.

Vitória et al. (2024) implemented a Multilayer Perceptron (MLP) to predict anxiety and depression scores based on responses to PROMIS^®^ questionnaires and self-reported wellbeing indicators (see Figure 3). The MLP architecture consisted of fully connected layers where each neuron applies a weighted sum of inputs followed by a non-linear activation. The forward propagation process is defined as in Equation 3:


(3) $$\begin{array}{l} h_{j}^{\left(i\right)}=f\left(\overset{n_{i-1}}{\underset{k=1}{\sum }}w_{k,j}^{\left(i-1\right)}h_{k}^{\left(i-1\right)}\right), \end{array}$$

where $h_{j}^{\left(i\right)}$ is the activation of the *j*-th neuron in the *i*-th hidden layer, $w_{k,j}^{\left(i-1\right)}$ is the weight between neuron *k* in the (*i*−1)-th layer and neuron *j* in the *i*-th layer, and *f* is the activation function. The model was trained using the Adam optimizer with a learning rate of 1 × 10^−4^, batch size of 128, and early stopping based on the number of epochs. A five-fold cross-validation was used for evaluation. Mohamed et al. (2023) developed a hybrid model integrating Support Vector Machines (SVM), Multilayer Perceptrons (MLP), and Random Forests. The MLP produced outputs via a nonlinear activation function, see Equation 4:


(4) $$\begin{array}{l} y=\Phi \left(w^{⊤}x+b\right) \end{array}$$

where Φ(·) is a nonlinear activation function. Thus, ANNs as a powerful tool for both research and clinical applications in mental health—particularly when combined with interpretability techniques that enhance transparency and build trust among practitioners.

**Figure 4:**
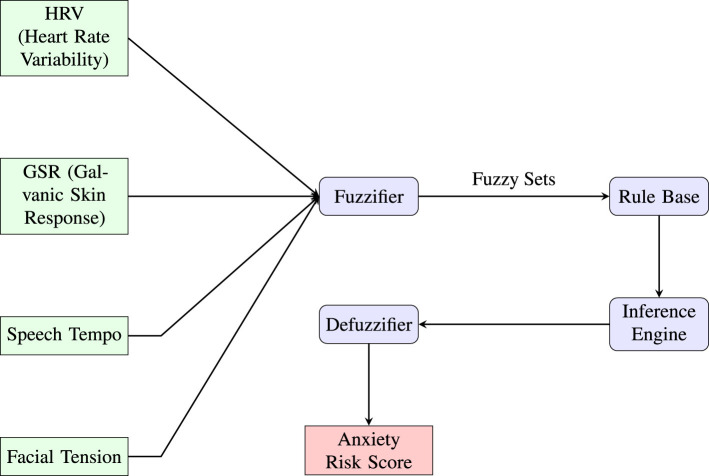
This diagram illustrates a fuzzy inference system (FIS) architecture designed for anxiety risk prediction using multimodal physiological and behavioral inputs. Key features such as heart rate variability (HRV), galvanic skin response (GSR), speech tempo, and facial tension are first mapped into fuzzy sets through the fuzzifier. These fuzzy representations are then processed by a rule base governed by expert-defined linguistic rules. The inference engine aggregates outcomes, which are then translated back into a crisp output via the defuzzifier, yielding an interpretable anxiety risk score. The modular design emphasizes explainability, enabling integration with clinical or wearable AI applications for mental health monitoring.

**Fuzzy Inference System** is another promising yet, less known approach to ML methods in predicting anxiety (see Figure 4). One type of Fuzzy Inference System are Neuro-Fuzzy Systems (NFS). NFS combine elements of neural networks and fuzzy logic. They can develop fuzzy rules and membership functions for input and output variables. NFSs are used for modeling complex and uncertain systems and find applications in various fields, including control systems, decision support, and pattern recognition.

The system processes inputs through five layers:

Layer 1 (Fuzzification): Each node computes a membership grade, see Equation 5:


(5) $$\begin{array}{l} O_{i}^{\left(1\right)}=\mu_{A_{i}}\left(x_{1}\right),\;O_{j}^{\left(1\right)}=\mu_{B_{j}}\left(x_{2}\right) \end{array}$$

Layer 2 (Rule Firing Strength): Each node multiplies incoming signals, see Equation 6:


(6) $$\begin{array}{l} w_{i}=\mu_{A_{i}}\left(x_{1}\right)\cdot \mu_{B_{i}}\left(x_{2}\right) \end{array}$$

Layer 3 (Normalization): Normalized firing strength, see Equation 7:


(7) $$\begin{array}{l} {\bar{w}}_{i}=\frac{w_{i}}{w_{1}+w_{2}} \end{array}$$

Layer 4 (Weighted Rule Output): Each node computes, see Equation 8:


(8) $$\begin{array}{l} {\bar{w}}_{i}f_{i}={\bar{w}}_{i}\left(p_{i}x_{1}+q_{i}x_{2}+r_{i}\right) \end{array}$$

Layer 5 (Output Aggregation): Final output, see Equation 9:


(9) $$\begin{array}{l} y=\underset{i}{\sum }{\bar{w}}_{i}f_{i} \end{array}$$

#### Gaussian process and general linear models

Portugal et al. (2019) applied Gaussian Process Regression (GPR) to predict anxiety levels based on fMRI activation patterns. GPR is appropriate for small sample sizes and high-dimensional fMRI data. They used both two-fold and five-fold CV with balanced scanner distributions across folds, applied 1,000 permutations and Bonferroni correction (threshold = 0.005), which is very cautious. Jafari et al. (2025) utilized Logistic Lasso Regression (LLR) and Logistic Elastic Net Regression (LENR) to classify anxiety in preschool children based on functional connectivity (FC) from fMRI data and clinical variables. LLR incorporates an L1 penalty to shrink coefficients of less important features to zero, effectively performing feature selection. LENR combines L1 and L2 penalties to handle correlated features and reduce model complexity. Perpetuini et al. (2021) employed a General Linear Model (GLM) for predicting state anxiety using physiological data such as pulse rate variability (PRV) and estimated systolic blood pressure (ABP).

### Other AI methods

Priya et al. (2020) compared five algorithms using DASS-21 scores, finding SVM most effective. Ku_Min_2024 evaluated five classifiers and introduced a composite score (CS) to assess model robustness. Bhatnagar et al. (2023) also employed Naive Bayes, which estimates class probabilities. Al-Nafjan and Aldayel (2024) employed three machine learning algorithms–Support Vector Machine (SVM), K-Nearest Neighbor (KNN), and Random Forest (RF)—for anxiety classification based on Galvanic Skin Response (GSR) signals. The SVM classifier separates classes by constructing an optimal hyperplane that maximizes the margin between different classes. The KNN classifier predicts class labels based on the majority class among the *k* closest samples.

**Figure 5:**
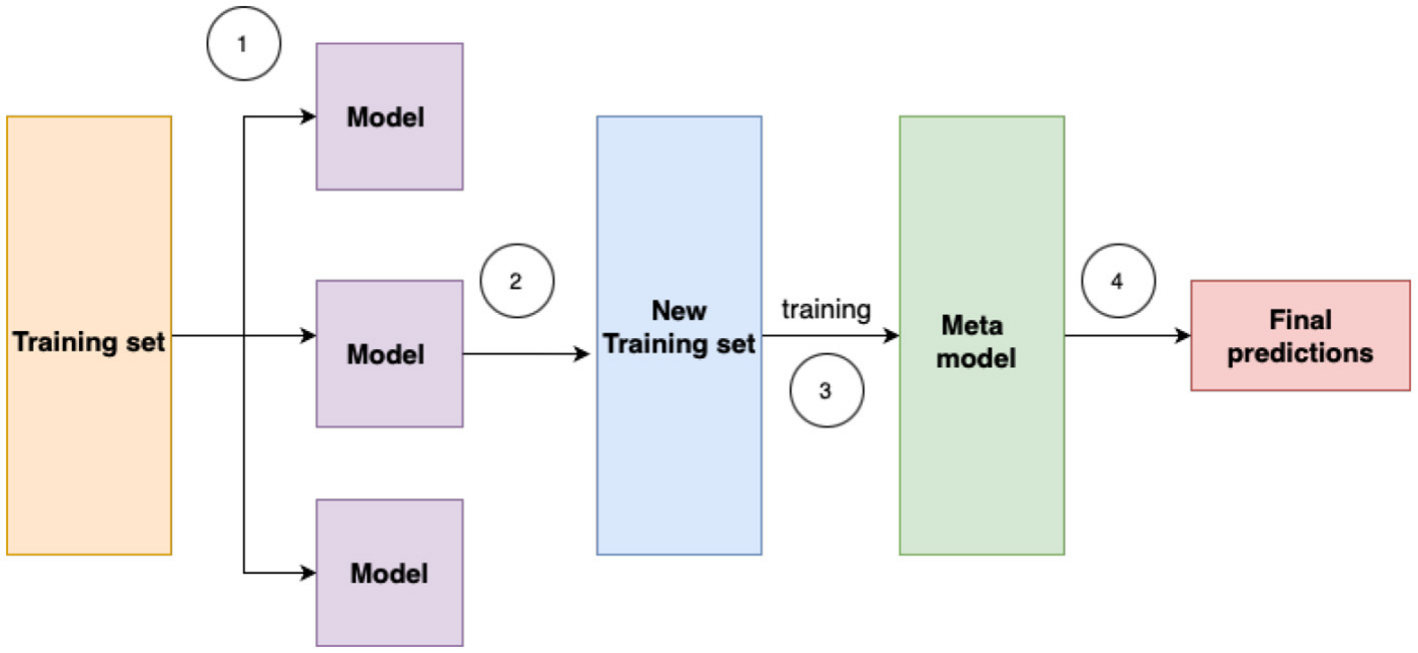
Stacking ensemble is an advanced machine learning technique that combines multiple base models to improve predictive performance. By training a meta-learner on the outputs of these base models, stacking leverages their complementary strengths and reduces generalization error.

**Ensemble methods** technique is another approach. They aim to improve the accuracy of results in models by combining multiple models instead of a single model. In machine learning models, errors often come from noise, variance, and bias sources. Ensemble methods in machine learning play a crucial role in minimizing these factors, ultimately improving the accuracy and stability of machine learning algorithms. They proved their effectiveness in reducing model variance, thereby increasing prediction accuracy. This reduction in variance occurs through the combination of multiple models, forming a single prediction selected from the various predictions generated by the ensemble of models. There are three types of ensemble methods: stacking, boosting, bagging. In Figure 5, the process of stacking is illustrated.

For example, Basterfield and Newman (2025) used Elastic Net and Gradient Boosted Trees to predict Generalized Anxiety Disorder (GAD). Daza et al. (2023) proposed a stacking ensemble approach using multiple base learners, with predictions aggregated by a level-1 meta-mode. Aldayel and Al-Nafjan (2024) utilized ensemble learning algorithms—Random Forest (RF), AdaBoost Bagging, and Gradient Bagging–to classify anxiety states from EEG signals.

**Multimodal learning with pretrained transformer and LSTM models** were used in Li et al. (2024). The researchers proposed a multimodal anxiety prediction model that integrates text and audio modalities. For the text modality, they employed BERT (Bidirectional Encoder Representations from Transformers) pretrained on Chinese corpora, fine-tuned on speech transcripts. For the audio modality, they used Long Short-Term Memory (LSTM) networks to extract temporal features such as pitch and Mel-Frequency Cepstral Coefficients (MFCCs). The output from both modalities was concatenated and passed to a linear classification layer for binary anxiety prediction (anxious vs. non-anxious). The model was evaluated using five-fold cross-validation, with early fusion yielding the best results.

### Anxiety screening methods

There are many methods currently used in the diagnosis of anxiety. The most used methods in the relevant literature include:

1. GAD-7: a questionnaire measuring generalized anxiety disorder severity. It has seven questions, with scores ranging from 0 to 21, with higher scores indicating more severe anxiety. It helps assess and monitor anxiety levels in clinical settings. It is widely used in studies by many researchers such as Daza et al. (2023), Qasrawi et al. (2022), and Bailey and Strunk (2023), etc., while some researchers utilized it as a base in building an original questionnaire.
2. DASS-21: stands for the Depression, Anxiety, and Stress Scale-21 Items. It is a self-report questionnaire designed to measure the severity of common mental health symptoms. The DASS-21 is a shorter version of the original DASS with 42 items. The questionnaire consists of three subscales, each focusing on a different aspect of mental health: depression, anxiety, and stress. Participants rate the extent to which they have experienced each symptom over the past week on a Likert scale. The DASS-21 is widely used in research and clinical settings to assess and monitor the emotional wellbeing of individuals. A study with the highest number of citations used it to screen anxiety and classify the severity (Priya et al., 2020). This research specifically identifies the consistency of optimal machine learning algorithms applicable across various scenarios, ultimately pinpointing Naive Bayes as the ideal choice.

## Results

### Dataset characteristics

The quality, diversity, and contextual relevance of datasets used in anxiety prediction research play a pivotal role in determining the robustness and generalizability of machine learning models. However, a review of current literature reveals recurring limitations in sample composition, data collection methodology, and transparency.

A major issue across studies is the reliance on relatively small and demographically narrow samples. For instance, Bhatnagar et al. (2023), Priya et al. (2020), Daza et al. (2023), Morr et al. (2024), and Vitória et al. (2024) used data collected from fewer than 400 participants, often drawn from specific university departments or regions, without stratifying for gender, socioeconomic status, or mental health history. Such constraints increase the risk of overfitting and restrict the applicability of models to broader populations. Even in studies with larger samples, such as Ku_Min_2024 (*N* = 4,184), the data was sourced from a single French university, thus limiting geographic and cultural diversity. An illustrative example is the study by Vitória et al. (2024), which collected longitudinal self-report data from 249 participants at a single Brazilian university using the PROMIS^®^ questionnaires. Although this study leveraged a rich feature set (80 variables) and repeated follow-ups (up to 15 responses per participant, totaling 1,924 records), the sample was restricted to university students, faculty, and staff aged 18 or older. While longitudinal designs enhance temporal understanding of mental health variation, the limited demographic scope and lack of clinical diagnosis constrain broader generalization.

Another concern lies in the age and educational homogeneity of participants. Studies like Benito et al. (2024), Basterfield and Newman (2025), and Li et al. (2024) primarily involved older adults or highly educated samples, while Portugal et al. (2019) and Perpetuini et al. (2021) included only healthy young adults. This creates a gap in model training data for mid-life or clinically diverse populations. Furthermore, some studies (e.g., Mohamed et al., 2023) did not clearly separate training features from outcome labels, introducing potential data leakage.

Sampling methods also varied in rigor. Several studies used convenience or snowball sampling via online forms (e.g., Qasrawi et al., 2022; Priya et al., 2020; Morr et al., 2024), which may result in self-selection bias and underrepresentation of vulnerable populations. In many cases, there was no mention of how missing data were handled (Qasrawi et al., 2022), nor were data distributions across anxiety severity levels provided.

Transparency in feature reporting was also inconsistent. While some studies provided full lists of variables used (e.g., Daza et al., 2023; Sau and Bhakta, 2017), others either omitted this information entirely or failed to report the wording of questionnaire items (Bhatnagar et al., 2023; Basterfield and Newman, 2025), making it difficult to replicate or extend the findings. In contrast, Vitória et al. (2024) explicitly detailed the feature importance using a connection weight algorithm, listing the top predictors for anxiety and depression, which enhances model interpretability and reproducibility.

Additional limitations emerge even in studies using relatively large or neurobiologically rich datasets. For example, Li et al. (2024) employed data from over 24,000 participants in the Canadian Longitudinal Study on Aging (CLSA), incorporating diverse predictors such as biological aging indices, personality traits, and mental health scales. However, the reliance on self-reported anxiety diagnoses and the relatively low recorded prevalence (2.93%) raise concerns about recall bias and underreporting. Similarly, Aldayel and Al-Nafjan (2024) utilized EEG data from only 23 participants exposed to anxiety-inducing stimuli, combining objective neurophysiological measures with subjective scales (HAM-A, SAM), but their small sample size and dependence on SMOTE for balancing may compromise external validity. Neuroimaging-based studies, like Jafari et al. (2025), also struggle with data constraints; despite using fMRI to analyze functional connectivity in preschool children, their final sample was limited to just 45 subjects after data cleaning, reducing statistical power and generalizability. Finally, even well-known public datasets such as WESAD, used by Al-Nafjan and Aldayel (2024), are constrained by small sample sizes (*N* = 15), male-skewed demographics, and lab-induced anxiety conditions that limit ecological validity. These examples collectively underscore the challenge of balancing dataset richness with representativeness, particularly in physiological and neurobiological anxiety prediction research.

While existing studies provide valuable insights into anxiety prediction, the datasets used often lack the heterogeneity, size, and documentation necessary for building scalable and clinically reliable models. These limitations highlight the need for more representative, longitudinal, and well-annotated datasets in future research efforts.

### Prediction models characteristics

This section summarizes various studies that utilize machine learning for predicting anxiety levels. The discussions encompass study outcomes, limitations, and future directions, offering insights into the achieved results, drawbacks encountered, and potential avenues for further exploration in this field. The key information for each study is presented in the Table 1. The Table 2 illustrates the distribution of machine learning method categories used across the reviewed studies on anxiety detection.

The mean classification accuracy across the selected studies is:


$$\bar{x}=\frac{1}{n}\overset{n}{\underset{i=1}{\sum }}x_{i}=\frac{1}{16}\overset{16}{\underset{i=1}{\sum }}x_{i}=83.45\%$$

The variance is:


$$\sigma^{2}=\frac{1}{n}\overset{n}{\underset{i=1}{\sum }}{\left(x_{i}-\bar{x}\right)}^{2}\approx 88.49$$

The standard deviation is:


$$\sigma =\sqrt{88.49}\approx 9.41$$

Table 2 summarizes and critically examines the limitations and constraints encountered in each study, such as sample size variability, data heterogeneity, model generalizability, and the interpretability of machine learning outputs in clinical contexts. Key information extracted from each study–including the nature of the sample, instruments used for anxiety assessment, machine learning algorithms implemented, participant numbers, parameter settings, and primary findings. This allows for a direct comparison of methodologies and outcomes across studies.

Table 1 emphasis is also placed on identifying trends in algorithm selection and the types of features most frequently associated with successful anxiety detection. It complements this by categorizing the machine learning methods employed, thereby offering a visual summary of the methodological landscape in this emerging area of research on anxiety detection.

**Table 1:** Summary of machine learning studies on anxiety detection with participant numbers and parameters.

**Study**	**Sample and instruments**	**ML methods**	**N-participants and parameters**	**Results**
Ögür et al. (2023)	Data of pregnant women	MPA and kNN combined	*N* = 393, kNN neighbors=5, window size = 2s	Accuracy = 98.11%
Portugal et al. (2019)	young adults, fMRI data	GPR	*N* = 154, GPR with 5-fold CV	*r* = 0.28
Ku and Min (2024)	University students	CNN, RF, XGBoost, LR, NB	*N* = 4,184, CNN layers = 3, max depth = 12	RF accuracy = 90%
Benito et al. (2024)	COVID-19 HADS survey (Spain)	RF, XGBoost, SVM, NB, MLP, LR	*N* = 9,291, MLP hidden layers = 2	AUROC = 0.72
Sau and Bhakta (2017)	HADS clinical data	BN, RF, MLP, J48, SMO, etc.	*N* = 520, multiple classifiers	RF accuracy = 89%
Basterfield and Newman (2025)	Diagnosed with GAD	GB trees, elastic net	*N* = 126, lambda grid search	Balanced accuracy = 72%
Perpetuini et al. (2021)	Healthy volunteers	GLM, supervised learning	*N* = 102 (54M/48F), age 20–70	*r* = 0.28
Fukazawa et al. (2019)	Smartphone logs + STAI	RF ensemble	*N* = 20, 10 decision trees	F-score = 74.2%
Mohamed et al. (2023)	Pre-clinical mental health	SVM, MLP, RF	*N* = 215, 80/20 split	RF = 98.13%
Qasrawi et al. (2022)	Arab countries dataset	GBM, DRF, ERF, etc.	*N* = 3,569, feature vector = 16-dim	GB = 82.9%, RF = 81.3%
Priya et al. (2020)	Online DASS-21	DT, RF, NB, SVM, KNN	*N* = 348, distance metric: Euclidean	NB accuracy = 73%
Daza et al. (2023)	GAD-7 online	Stacking 1A-4A	*N* = 284, meta-learner: LR	Stacking 4A = 97.83%
Bhatnagar et al. (2023)	GAD-7 and DASS-21	NB, DT, RF, SVM	*N* = 127, 5-fold CV	RF = 78.9%
Vitória et al. (2024)	Self-report data + air quality	MLP, RF, GB, SVM, DT	*N* = 249, MAPE loss function	RF: MAPE = 6.31%
Li et al. (2024)	CLSA dataset	RF with undersampling	*N* = 24,388 (714 anxiety), resampling strategy	Balanced accuracy = 74%
Jafari et al. (2025)	Preschool MRI data	LLR, LENR	*N* = 45, regularization λ tuned	LLR accuracy = 78%
Aldayel and Al-Nafjan (2024)	DASPS EEG, DWT/PSD	Ensemble, KNN, LDA, SVM	*N* = 249, KNN *k* = 3, SVM RBF kernel	RF accuracy = 87.5%
Al-Nafjan and Aldayel (2024)	GSR from WESAD	SVM, KNN, RF	*N* = 15, 14-layer autoencoder	KNN: 98.2%, classic: 96.9%
Morr et al. (2024)	Lebanese universities, COVID + lifestyle	LR, MLP, SVM, RF, etc.	*N* = 329, 9 classifiers, 70/30 split	NB: AUC = 76.37%

**Table 2:** Categorized ML methods across studies.

**Study**	**SVM**	**kNN**	**Neural Nets**	**Trees**	**Linear**	**Probabilistic**	**Ensemble**
Ögür et al. (2023)		✓					
Portugal et al. (2019)						✓	
Ku and Min (2024)				✓	✓		✓
Benito et al. (2024)	✓		✓		✓		✓
Sau and Bhakta (2017)			✓	✓	✓	✓	
Basterfield and Newman (2025)					✓		✓
Perpetuini et al. (2021)					✓		
Fukazawa et al. (2019)							✓
Mohamed et al. (2023)	✓		✓				✓
Qasrawi et al. (2022)	✓		✓	✓			✓
Priya et al. (2020)	✓	✓		✓		✓	✓
Daza et al. (2023)							✓
Bhatnagar et al. (2023)	✓			✓		✓	✓
Vitória et al. (2024)	✓		✓	✓			✓
Li et al. (2024)					✓		✓
Jafari et al. (2025)			✓				
Aldayel and Al-Nafjan (2024)	✓	✓			✓		✓
Al-Nafjan and Aldayel (2024)	✓	✓					✓
Morr et al. (2024)	✓	✓	✓		✓	✓	✓
**Total**	**9**	**6**	**6**	**6**	**7**	**6**	**14**

### Review of machine learning for anxiety prediction

#### ML for anxiety prediction in geriatric and general populations

Sau and Bhakta (2017) researched predicting anxiety and depression in elderly patients using ML algorithms. They evaluated 520 geriatric patients using the Hospital Anxiety and Depression Scale (HADS). Ten ML algorithms were assessed, including Random Forest, Bayesian Network, Logistic Regression, Multiple Layer Perception, Naive Bayes, and others. Random Forest demonstrated the highest accuracy at 89% using 10-fold cross-validation. Predictors such as age, sex, marital status, socio-economic conditions, family environment, literacy, job security, history of depression, and chronic medical conditions were considered. Priya et al. (2020) aimed to identify the most suitable ML algorithm for predicting anxiety, depression, and stress using the DASS-21 questionnaire with 348 participants aged 20–60. They employed Decision Tree, Random Forest Tree, Naive Bayes, Support Vector Machine, and KNN algorithms. The study revealed Naive Bayes achieved a high accuracy of 73.3%, while Random Forest emerged as the best model based on the f1 score, highlighting the importance of considering metrics beyond accuracy, especially with imbalanced classes. Key items of a questionnaire such as “scared_without_any_good_reason,” “Life_was_meaningless,” and “Difficult_to_relax” were identified as significant for predicting Anxiety, Depression, and Stress, respectively. This underscores the relevance of specific indicators for each mental health dimension. Qasrawi et al. (2022) investigated efficient algorithms for anxiety and depression prediction among pregnant and postpartum women using a dataset from five Arab countries during the COVID-19 pandemic. They included 3 569 participants and assessed seven ML algorithms, and found Gradient Boosting (GB) and Random Forest (RF) excelled, achieving 82.9 and 81.3% accuracy for anxiety and 83.3 and 83.2% for depression, respectively. Drawbacks included a relatively small sample size and the subjective nature of online self-reported assessments. To enhance future research, the study suggests expanding efforts with larger, more representative datasets that include clinical information, especially in low- and middle-income countries, to improve generalizability. In Bhatnagar et al. (2023), a study involving 127 students in India, they used a questionnaire based on GAD-7 and DASS-21 and applied Naive Bayes, Decision Tree, Random Forest, and SVM algorithms to classify anxiety levels. The ML algorithms achieved an average accuracy of 75%, with Random Forest leading at 78.9%. Their research aimed to uncover the causes and effects of anxiety among Indian students, measure anxiety levels using their questionnaire, and determine the most effective ML algorithm for classification. Results indicated that as causes of anxiety increase, the corresponding effects worsen, offering insights for potential mitigation strategies.

Additionally, the questionnaire reliably measured anxiety levels, while Random Forest emerged as the best classifier with 78.9% accuracy. For future endeavors, they suggested expanding the research across various academic departments and locations within India to comprehend the regional impacts and diverse challenges students face. This broader scope aims to tailor interventions more effectively to cater to the specific needs of different student subsets. Mohamed et al. (2023) examined anxiety stages in a dataset of 215 individuals using SVM, MLP, and RF models. RF achieved 98.13% accuracy in predicting minimal to severe anxiety stages. Limitations included the absence of biological markers, a small sample size, and the “black box” nature of ML algorithms in mental health. “Black box” means that ML algorithms often lack transparency as there are difficulties in understanding how these algorithms generate their recommendations or predictions. Because of that, people may not have much trust in the results of techniques, especially when dealing with sensitive issues, such as mental health. The study proposed a hybrid model prediction and recommendation model based on anxiety prevalence. It suggests the potential for broader data inclusion in complex contexts like conflict zones for improved diagnoses. In Li et al. (2024), researchers used Random Forest with Under-Sampling (RF-US) to prospectively predict anxiety onset approximately 3 years later among 24,388 middle-aged and older Canadian adults from the CLSA dataset. The study included 2,599 predictors covering demographics, personality traits, frailty, and mental health indicators. Shapley values highlighted significant predictors such as prior depression or mood disorders, frailty, anxious personality, and emotional instability. The model achieved an AUC of 0.814 ± 0.016, balanced accuracy of 74.1%, sensitivity of 74.3%, and specificity of 73.8%, demonstrating strong predictive capacity. This study illustrates machine learning's potential for early detection of anxiety onset, which can support preventive interventions in aging populations.

#### Smartphone-based behavioral monitoring for anxiety

Fukazawa et al. (2019) explored using smartphone logs and anxiety state assessments to predict anxiety levels. They employed Random Forest, an ensemble of decision trees, and proposed a method combining environmental, real-world, and online behavioral aspects into categorical features. This approach achieved a high F-score of 74.2%, surpassing the existing method by 4.0%. The study aimed to correlate smartphone data with anxiety levels, using STAI questionnaires to detect anxiety states. The method's success suggests that combining environmental, real-world behavioral, and online behavioral features can accurately predict anxiety levels, highlighting the importance of smartphone usage patterns, especially viewing smartphones in dark places, in detecting increases in anxiety. Additionally, the study proposed the potential for measuring stress levels without self-assessment by leveraging combined smartphone-acquired features. In Daza et al. (2023), a study targeting anxiety levels among college students using the GAD-7 questionnaire with 284 participants, they proposed and compared four combined models based on Stacking techniques. Their best-performing model, Stacking 4A: KNN-Ensemble, achieved a high accuracy of 97.83% by employing data oversampling for balancing. They concluded that the combined approach outperformed individual algorithms, effectively predicting anxiety levels. However, the study acknowledged limitations, suggesting the consideration of more comprehensive assessment tools like Dass 21 for a detailed analysis of other mental health disorders beyond anxiety, such as stress and depression. Additionally, they proposed the creation of a new test incorporating environmental, social, and socio-economic factors for a more holistic analysis of anxiety levels. In Benito et al. (2024), researchers analyzed data from 9,291 individuals in Northern Spain using socioeconomic, demographic, lifestyle, and COVID-19-specific factors to predict anxiety, depression, and self-perceived stress. Utilizing machine learning algorithms (Random Forest, XGBoost, SVM, Naive Bayes, Multi-layer Perceptron, and Logistic Regression), XGBoost emerged as the highest-performing model, achieving AUROC scores of 0.78 for depression, 0.72 for anxiety, and 0.74 for self-perceived stress. Binary classification models (healthy vs at-risk) showed high precision and recall, making them effective screening tools. Important predictors were poor self-reported health status, chronic mental conditions, and lack of social support. A novel combination of SHAP and UMAP was used to explain the models' predictions and effectively identify high-risk phenotypic clusters, aiding targeted intervention strategies. The authors recommended further validation in broader populations and emphasized the approach's potential to enhance public health preparedness and tailored mental health interventions during emergencies.

#### Neuroimaging and physiological signal-based models

In Portugal et al. (2019), a study involving 154 young adults (103 females) with various levels of psychological distress, including subthreshold cases and diagnosed psychiatric disorders, utilized functional MRI data during a dynamic emotional face processing task. Gaussian Process Regression (GPR) was applied to predict anxiety and depression symptoms measured by self-report and clinician-rated scales (STAI-T, STAI-S, MASQ-D, HAM-A, HDRS). The models significantly predicted trait anxiety (STAI-T) scores with an average correlation (*r*) of 0.28 and mean squared error (MSE) around 4.5 using two-fold and five-fold cross-validation. Findings indicated neural responses to emotional faces correlated with anxiety severity along a continuum, rather than distinct categorical diagnoses. Predictive brain patterns involved subtle, distributed activation across various cortical regions rather than localized regions, suggesting a dimensional and multivariate approach may effectively inform clinical assessment and interventions. The authors recommended extending this approach to larger, multi-dimensional studies to further refine clinical prediction models based on neuroimaging. Perpetuini et al. (2021) explored the prediction of state anxiety using physiological features extracted from photoplethysmography (PPG) signals and a multivariate machine learning model. The study included 102 healthy participants (54 males, 48 females; age range: 20–70 years, M = 34.3, SD = 15.5) who avoided stimulants prior to the experiment. Using features like RMSSD and LF/HF, a Generalized Linear Model (GLM) was trained to predict STAI-Y anxiety scores, achieving a strong correlation (*r* = 0.81). Gender was also found to be a relevant predictor. The findings demonstrate the potential of PPG and machine learning for real-time anxiety monitoring in affective computing and human-robot interaction contexts. In Xu et al. (2024), authors examined the relationship between neuroanatomical brain features and anxiety trajectories in adolescence using logistic regression. Drawing on data from 4,119 participants in the Adolescent Brain Cognitive Development (ABCD) study, the authors extracted cortical thickness and surface area measurements across 68 brain regions. Participants were categorized into high- or low-anxiety trajectory groups based on longitudinal anxiety symptom patterns assessed over 2 years. Logistic regression models trained on surface area features achieved the highest performance, with an AUC of 0.74 and accuracy of 70%. These findings suggest that neuroanatomical features may serve as early biomarkers for identifying youth at risk of chronic anxiety. In Jafari et al. (2025), researchers aimed to identify functional MRI (fMRI) biomarkers and evaluate machine learning methods to diagnose anxiety in preschool children using emotional face tasks. Forty-five preschool children from the Duke Preschool Anxiety Study underwent fMRI scans under angry and fearful face conditions. Functional connectivity (FC) between limbic system regions was computed and combined with demographic and clinical data to classify anxiety using Logistic Lasso Regression (LLR) and Logistic Elastic Net Regression (LENR). The LLR model performed optimally, achieving accuracies of 78% (angry) and 78% (fearful), highlighting specific connectivity biomarkers such as medial prefrontal cortex (MPFC) connectivity patterns as predictive features. This approach demonstrates promise in using neuroimaging biomarkers for early anxiety diagnosis. In Basterfield and Newman (2025), researchers used machine learning to predict long-term recovery in 126 individuals with Generalized Anxiety Disorder (GAD), using demographic, clinical, psychological, biological, and lifestyle data collected at baseline. Two machine learning models were tested: Gradient Boosted Trees and Elastic Net Logistic Regression. The Elastic Net model showed superior performance with an AUC of 0.81 and balanced accuracy of 72% (sensitivity = 0.70, specificity = 0.76), effectively predicting GAD recovery 9 years later. Important predictors of non-recovery included higher depressed affect, frequent experiences of daily discrimination, and more visits to healthcare providers. Conversely, predictors of recovery encompassed higher education, older age, stronger friend support, higher waist-to-hip ratio, and greater positive affect. The findings provide critical insights into factors influencing long-term anxiety outcomes and suggest that Elastic Net models are valuable tools for tailoring interventions and improving prognosis in individuals with GAD.

#### Deep learning and robust neural approaches

In Vitória et al. (2024), researchers applied machine learning models, primarily Multi-Layer Perceptron (MLP), to predict anxiety and depression scores using PROMISⓇ questionnaire data collected from 249 participants at a Brazilian university. The dataset included 80 variables, capturing symptoms, general wellbeing, and environmental perceptions over a three-month follow-up period. Among multiple models tested (RF, SVM, DT, GB, and MLP), the MLP model demonstrated superior performance, achieving an average MAPE of 6.31%, *R*^2^ of 0.76, and a Spearman correlation of 0.8886 (or 0.89). These findings highlight the efficacy of neural network models in capturing complex mental health patterns and underscore the significance of subjective wellbeing and symptom perception variables in predicting anxiety and depression. In Ku and Min (2024), a study involving 4,184 undergraduate students from the University of Nice Sophia Antipolis, France, utilized demographic, biomedical, and self-reported survey data to evaluate the predictive capabilities of machine learning algorithms for Major Depressive Disorder (MDD) and Generalized Anxiety Disorder (GAD). Five ML techniques–CNN, Random Forest, XGBoost, Logistic Regression, and Naive Bayes–were employed. CNN exhibited superior performance and robustness when faced with subjective response errors, maintaining high accuracy, Cohen's kappa, and positive precision. Random Forest also demonstrated significant precision in correctly identifying individuals with these conditions. Physiological markers (e.g., heart rate, blood pressure) and environmental factors (e.g., parental home environment) emerged as significant predictors. The study underscores the importance of algorithmic resilience and recommends CNN as particularly effective in scenarios involving unreliable subjective data. In Aldayel and Al-Nafjan (2024), researchers explored EEG-based anxiety detection using machine learning and ensemble learning techniques on the DASPS dataset comprising EEG signals from 23 participants exposed to anxiety-provoking stimuli. EEG features were extracted using discrete wavelet transform (DWT) and power spectral density (PSD), labeled with Hamilton Anxiety Rating Scale (HAM-A) and Self-Assessment Manikin (SAM). Various classifiers, including Random Forest (RF), AdaBoost Bagging, Gradient Bagging, KNN, LDA, and SVM were evaluated. The Random Forest classifier with DWT-based features and HAM-A labeling achieved the highest accuracy (87.5%), precision (87.65%), and recall (87.5%), outperforming other classifiers. This study highlights the effectiveness of ensemble methods and EEG signal processing techniques for reliable anxiety detection.

#### Comparative algorithm studies and metric evaluation

In Al-Nafjan and Aldayel (2024), the authors developed an anxiety detection system using Galvanic Skin Response (GSR) signals and machine learning methods. They analyzed the WESAD dataset, extracting features through traditional statistical methods and an automatic approach employing a 14-layer autoencoder. Three classifiers–Support Vector Machine (SVM), K-Nearest Neighbor (KNN), and Random Forest (RF)–were evaluated. The KNN algorithm achieved the highest performance, attaining accuracy rates of 98.2% with automatic feature extraction and 96.9% with statistical methods. This research highlights the potential of advanced feature extraction techniques and emphasizes the robustness of GSR signals combined with KNN for accurate anxiety detection.

### Evaluation metrics for assessing the precision of anxiety measurements

Evaluation metrics play a crucial role in determining the precision and reliability of anxiety prediction models, guiding researchers in assessing model performance and clinical applicability. Table 3 presents the metrics used to assess students' anxiety and stress using prediction models. Based on this, the most frequently used metrics were Accuracy (Bhatnagar et al., 2023; Priya et al., 2020; Daza et al., 2023; Sau and Bhakta, 2017; Mohamed et al., 2023; Qasrawi et al., 2022; Basterfield and Newman, 2025; Ku and Min, 2024; Li et al., 2024; Jafari et al., 2025; Aldayel and Al-Nafjan, 2024; Al-Nafjan and Aldayel, 2024; Morr et al., 2024), which measures the overall correctness of a model, and F1 Score (Priya et al., 2020; Daza et al., 2023; Fukazawa et al., 2019; Qasrawi et al., 2022; Benito et al., 2024; Ku and Min, 2024; Al-Nafjan and Aldayel, 2024; Morr et al., 2024), which balances precision and recall and is particularly useful with imbalanced datasets.

**Table 3:** Performance metrics and their corresponding methodological references used in evaluating machine learning models across clinical and mental health datasets.

**Study**	**Accuracy**	**Precision**	**Recall**	**Specificity**	**F1**	**AUC**	**Sensitivity**	**MAPE**	**Error rate**	**RMSE**
Bhatnagar et al. (2023)	✓									
Priya et al. (2020)	✓	✓	✓	✓	✓				✓	
Daza et al. (2023)	✓			✓	✓		✓		
Sau and Bhakta (2017)	✓									
Mohamed et al. (2023)	✓									✓
Qasrawi et al. (2022)	✓	✓	✓	✓	✓					
Basterfield and Newman (2025)	✓			✓		✓	✓			
Ku and Min (2024)	✓	✓	✓		✓	✓			✓	
Li et al. (2024)	✓			✓		✓	✓			
Jafari et al. (2025)	✓	✓	✓			✓				
Aldayel and Al-Nafjan (2024)	✓	✓	✓			✓				
Al-Nafjan and Aldayel (2024)	✓	✓	✓		✓					
Morr et al. (2024)	✓	✓	✓	✓	✓	✓	✓			
Portugal et al. (2019)										✓
Benito et al. (2024)		✓	✓		✓					
Fukazawa et al. (2019)					✓		✓			
Perpetuini et al. (2021)	✓									
Vitória et al. (2024)								✓		
**Total**	**13**	**8**	**8**	**6**	**8**	**7**	**5**	**1**	**2**	**2**

These metrics offer perspectives on model performance and are selected based on the specific diagnostic goals and characteristics of the class imbalance.

Some studies (e.g., Perpetuini et al., 2021) reported AUC but omitted essential classification metrics such as F1-score and sensitivity, which are crucial in clinical screening contexts. Similarly, Vitória et al. (2024) focused on regression-based metrics (MAPE, *R*^2^, and Spearman correlation) to assess anxiety and depression scores, but did not report classification-specific measures such as accuracy or F1-score. While this is appropriate given their continuous outcome variables, it complicates the comparability with studies using classification tasks and limits insights into clinical decision thresholds. Additionally, several papers, including Daza et al. (2023) and Priya et al. (2020), relied on oversampling techniques without reporting class-specific performance, raising concerns about potential overfitting in minority anxiety classes. In contrast, only a few studies, like Qasrawi et al. (2022), reported multiple metrics including MCC and F1-score, providing a more comprehensive evaluation. These inconsistencies highlight the need for a standardized evaluation framework, especially in mental health prediction, where dataset imbalance and false negatives can have critical implications.

### Variable selection methods used for anxiety prediction

The variable selection process plays a critical role in refining model performance, reducing overfitting, and identifying features that are most predictive of anxiety-related outcomes. In the reviewed literature, a variety of selection techniques were used, although their application varied considerably in transparency and rigor. The Table 4 presents the studies and the variable selection methods that they used.

**Table 4:** Variable selection techniques and their corresponding references commonly used in clinical and behavioral data analysis.

**Study**	**None**	**CFS**	**RF**	**XGB**	**Chi2**	**IG**	**mRMR**	**PLR**	**CWA**	**SHAP**	**LLR/LNR**
Bhatnagar et al. (2023)	✓										
Daza et al. (2023)	✓										
Qasrawi et al. (2022)	✓										
Priya et al. (2020)	✓										
Portugal et al. (2019)	✓										
Ku and Min (2024)	✓										
Aldayel and Al-Nafjan (2024)	✓										
Al-Nafjan and Aldayel (2024)	✓										
Sau and Bhakta (2017)		✓									
Fukazawa et al. (2019)			✓	✓							
Morr et al. (2024)			✓								
Sahu et al. (2023)					✓						
Mohamed et al. (2023)						✓					
Benito et al. (2024)							✓				
Basterfield and Newman (2025)								✓			
Vitória et al. (2024)									✓		
Li et al. (2024)										✓	
Jafari et al. (2025)											✓
**Total**	**8**	**1**	**2**	**1**	**1**	**1**	**1**	**1**	**1**	**1**	**1**

These methods–ranging from filter approaches like mutual information to embedded techniques such as LASSO–help reduce model complexity, enhance interpretability, and improve generalization by identifying the most relevant predictors.

Some studies employed formal feature selection methods. For instance, Daza et al. (2023) utilized Information Gain to rank input variables before feeding them into stacking models, while Mohamed et al. (2023) selected top predictors using filter-based techniques. Sau and Bhakta (2017) tested five different selection methods (CFS, PCA, Relief, Gain Ratio, and Info Gain) before selecting a final feature set, which enhanced interpretability and minimized redundant inputs. Similarly, Ku and Min (2024) used mRMR (Minimum Redundancy Maximum Relevance) to filter features from an initial set of 161 to improve model generalizability. These approaches demonstrate an awareness of the importance of input optimization.

A notable example of model interpretability is found in Vitória et al. (2024), who applied a connection weight algorithm to quantify the relative importance of each input variable in their MLP-based regression model. This method calculated the contribution of each input node based on learned weights, providing transparency into which PROMIS^®^ questionnaire items and self-reported symptoms most influenced anxiety and depression predictions. Features such as emotional distress, fatigue, and perceived social functioning were identified as the most influential. Although the study did not apply a feature reduction step before training, the *post hoc* analysis of feature importance helped clarify the model's decision-making process.

However, in several other studies, variable selection was either underreported or completely omitted. For example, Priya et al. (2020) and Bhatnagar et al. (2023) relied solely on DASS-21-derived responses or custom questionnaires, yet did not disclose whether or how any feature reduction was applied. In these cases, it remains unclear whether all questionnaire items were used or whether multicollinearity and redundancy were considered–raising concerns about possible noise retention and overfitting. Additionally, Mohamed et al. (2023) used GAD-7 both to derive input variables and classify output labels, potentially introducing data leakage and undermining the validity of feature importance analysis. However, some studies did not use variable selection methods, but instead relied on domain knowledge and previously validated EEG techniques (Aldayel and Al-Nafjan, 2024). Furthermore, embedded feature selection was indirectly performed via ensemble methods, particularly Random Forest, inherently selecting important features by emphasizing informative features during tree construction. Some studies, such as Aldayel and Al-Nafjan (2024) and Al-Nafjan and Aldayel (2024) did not use explicit external feature selection methods (e.g., recursive feature elimination or SHAP), but used alternatives like ensemble techniques (Aldayel and Al-Nafjan, 2024), traditional statistical extraction (Al-Nafjan and Aldayel, 2024), Random Forest (Morr et al., 2024).

Furthermore, some models, such as those in Benito et al. (2024) and Qasrawi et al. (2022), included a wide array of sociodemographic and lifestyle variables, but lacked a clear methodological explanation of how input features were selected or filtered. The absence of such documentation hinders reproducibility and raises questions about model robustness, especially when applied to new populations.

Overall, the inconsistent application and reporting of feature selection methods highlight the need for clearer standards in anxiety prediction research. Models that omit or insufficiently describe this process may suffer from reduced generalizability and increased risk of overfitting, particularly when working with small or imbalanced datasets.

## Discussion

The integration of machine learning into mental health research offers unprecedented opportunities to improve the prediction, diagnosis, and treatment of anxiety disorders, yet this rapidly evolving field remains hindered by critical methodological challenges and gaps in clinical applicability. The current study systematically examined current machine learning approaches in anxiety research, identifying recurring limitations such as small and homogeneous datasets, inconsistent evaluation metrics, and a lack of model interpretability, ultimately emphasizing the urgent need for standardized methodologies and explainable AI to enhance both scientific rigor and clinical trust.

The primary research question guiding this systematic review was: (1) To what extent has machine learning (ML) been used to predict anxiety? Our analysis reveals a growing body of work applying ML to classify anxiety status or estimate severity based on diverse data modalities, including self-report instruments (e.g., GAD-7, DASS-21), physiological signals (e.g., heart rate variability, electrodermal activity), and behavioral patterns. However, the field remains fragmented. Most studies rely on cross-sectional or small-scale datasets, limiting generalizability. There is also a lack of methodological consistency, and longitudinal or prospective models capable of predicting future anxiety onset remain notably scarce.

The secondary research question explored: (1.1) What ML methods have been applied, and which have demonstrated superior performance? Across the literature, ensemble techniques such as Random Forests and Gradient Boosting consistently yield high predictive accuracy (often exceeding 95%). Support Vector Machines (SVMs) perform robustly with high-dimensional data, while K-Nearest Neighbors (KNN) and Stacking ensembles have also shown promise. Neural network-based approaches, including Artificial Neural Networks (ANNs) and Multilayer Perceptrons (MLPs), are well-suited for capturing nonlinear relationships, particularly in multimodal datasets.

An additional research question examined: (1.2) Which feature selection algorithms are commonly used? Commonly reported techniques include Recursive Feature Elimination (RFE), Principal Component Analysis (PCA), and embedded methods such as L1 regularization or tree-based feature importance. However, many studies fail to explicitly describe feature selection procedures, undermining model interpretability and reproducibility.

Finally, we addressed: (1.3) What evaluation metrics are used to assess the performance of ML models in anxiety prediction? Accuracy is the most frequently reported metric. However, F1 Score and Area Under the ROC Curve (AUC-ROC) are increasingly utilized, particularly in the context of class imbalance. Additional metrics such as Precision, Recall, and Mean Squared Error (MSE) also appear in some studies. Nonetheless, external validation is rarely conducted, posing a barrier to clinical translation and deployment.

### Strengths of AI methods in anxiety prediction

The current application of AI in anxiety prediction and diagnosis exhibits several promising strengths. First, machine learning models can process and integrate large, complex datasets–ranging from clinical assessments to digital behavioral data such as smartphone usage and physiological signals–enabling richer, multidimensional insights beyond traditional methods. This capacity facilitates earlier and potentially more accurate identification of anxiety symptoms, even before clinical presentation. Second, AI algorithms have demonstrated the ability to uncover subtle patterns and biomarkers associated with anxiety disorders that may be imperceptible to human clinicians, enhancing precision in diagnosis and personalized treatment planning. Techniques such as natural language processing (NLP) applied to patient narratives or social media posts allow non-invasive monitoring of mental health trends in real-time. Third, the scalability and efficiency of AI-driven tools offer great potential for extending mental health services to underserved populations, addressing resource constraints and improving accessibility. For instance, digital screening apps powered by AI can facilitate continuous, remote anxiety monitoring, which is especially valuable in contexts with limited mental health professionals. Lastly, advancements in explainable AI and model interpretability are beginning to address previous transparency concerns, allowing clinicians to better understand and trust AI recommendations, which is crucial for integration into routine care. Together, these strengths underscore AI's transformative potential in anxiety research, diagnosis, and intervention, even as the field continues to address remaining challenges.

A further constraint is the limited ecological validity of current studies. Most multimodal systems are validated in lab-controlled settings with low environmental variability and narrow population samples–often university students or clinical volunteers. Real-world deployment would require addressing hardware constraints (e.g., wearable sensor stability, smartphone microphone fidelity), minimizing signal dropout, and improving preprocessing pipelines to handle artifacts and missing data. Additionally, assembling diverse, large-scale datasets that span race, age, socioeconomic background, and health conditions is critical to building generalizable models (Razavi et al., 2023).

Beyond performance, multimodal fusion also meaningfully contributes to model interpretability, which is critical for clinical application. By triangulating across data modalities, clinicians can gain a more holistic view of a patient's mental state and better understand the model's rationale. In explainable AI (XAI) frameworks, modalities can be independently analyzed using tools such as SHAP (Shapley Additive Explanations), LIME, or attention maps to identify which features drove a specific prediction. For instance, an anxiety alert may be triggered due to a combination of increased speech tempo, elevated heart rate, and reduced lexical diversity–patterns that align with known clinical markers (Lakomkin et al., 2021). This disaggregation enables transparent dialogue between AI and clinician, promoting trust, accountability, and ethically sound integration into care workflows. Moreover, recent developments in transformer-based models and self-supervised learning present new opportunities for multimodal learning without the need for vast labeled datasets. Frameworks like MM-BERT and UniT leverage cross-modal attention to learn shared representations from sparse or weakly labeled data, which could be pivotal in mental health applications where annotation is costly and subjective (Tsai et al., 2019). These architectures not only improve robustness but also provide modular interpretability, where individual attention heads can be assigned to specific modalities for transparent inference tracking.

In summary, integrating multimodal data into anxiety prediction frameworks represents a tangible advancement in building robust, sensitive, and trustworthy AI systems. However, realizing this potential requires standardizing fusion strategies, improving dataset diversity, and expanding validation beyond lab settings. Multimodal fusion, when implemented thoughtfully, bridges the gap between complex human affect and computational representations, and holds promise for more accurate, accessible, and ethical mental health care - promising for the future work.

### Multimodal fusion systems

In addition to the limitations identified, one promising yet underexplored direction in anxiety prediction research is the use of multimodal fusion techniques–the integration of multiple data types such as text, audio, and physiological signals. Multimodal approaches hold the potential to enrich ML models by capturing diverse dimensions of anxiety expression. For example, while self-reported textual input or linguistic cues from social media can reflect cognitive aspects of anxiety, physiological data (e.g., electrodermal activity, heart rate variability) provide insights into somatic arousal, and vocal features (e.g., pitch, tone) can signal emotional instability. Integrating these sources allows models to learn richer, temporally synchronized representations that mirror the multifaceted nature of anxiety more closely than unimodal inputs alone (Ancillon et al., 2022). Recent reviews, such as that of Ancillon et al. (2022), underscore the increasing viability of multimodal systems, particularly when sensor data (e.g., ECG, EEG, GSR) is combined with behavioral inputs (e.g., text, facial microexpressions) and paralinguistic features (e.g., vocal tone and prosody). These approaches have demonstrated superior diagnostic capabilities in detecting anxiety, depression, and stress-related disorders, often achieving classification accuracies above 90% in controlled environments. For example, Mishra and Lin (2021) reported significant improvement in detection performance when fusing electrodermal activity with facial expression cues using deep neural networks.

Despite these promising outcomes, key technical and practical challenges remain. Synchronizing heterogeneous data streams across different temporal resolutions is nontrivial; behavioral signals like speech and typing cadence are challenging with physiological rhythms. This demands sophisticated time-alignment mechanisms, dynamic windowing, or attention-based architectures to retain signal coherence. Furthermore, the field lacks consensus on fusion strategy taxonomy–i.e., whether features should be combined at raw input (early fusion), representation (intermediate fusion), or decision level (late fusion). While early fusion offers the potential for richer representations, it is more sensitive to noise and missing modalities; in contrast, late fusion sacrifices some nuance but offers modularity and robustness (Baltrušaitis et al., 2019).

Overall, integrating ML systems into real-world clinical workflows involves challenges of trust, regulatory approval, and model drift over time. Anxiety prediction systems must be adaptive to evolving behavioral patterns, yet stable enough for long-term clinical use. Addressing these challenges will require collaboration between data scientists, clinicians, and ethicists, as well as advances in privacy-preserving ML and federated learning paradigms.

### Critical analysis

One major limitation in current mental health research employing machine learning (ML) is the often opaque or “black-box” nature of these algorithms. As highlighted by Mohamed et al. (2023), many ML models operate in ways that are difficult for researchers and clinicians to interpret, making it challenging to understand the rationale behind specific predictions. This lack of transparency can significantly undermine confidence in the outcomes generated by these systems, particularly when applied to sensitive and high-stakes areas such as mental health diagnosis and treatment. Trust and interpretability are crucial for clinical adoption, and their absence poses a serious barrier to integrating ML tools effectively in practice.

Another critical issue concerns the limited sample sizes used in many studies, which restricts the generalizability and robustness of findings. As shown in Table 1, only two studies analyzed included more than 1,000 participants, while the average sample size across others hovers around 270. Such small datasets increase the risk of overfitting and reduce statistical power, ultimately compromising the reliability of the models. Additionally, certain studies, including Bailey and Strunk (2023) and Mohamed et al. (2023), fail to report key performance metrics like specificity–the algorithm's ability to correctly identify negative cases (i.e., patients without the disorder). Without this information, it is difficult to fully assess the clinical utility and safety of these ML approaches. The reviewed studies collectively illustrate a clear and growing interest in leveraging machine learning techniques for predicting anxiety. However, a number of recurring methodological and practical limitations hinder the progress and applicability of this research.

### Related work

The landscape of machine learning (ML) applications in mental health has seen the emergence of several secondary studies that synthesize existing evidence, providing valuable insights into methodological trends and challenges. Influential recent works include systematic reviews and surveys by Schaab et al. (2024), Ancillon et al. (2022), and Razavi et al. (2023).

Schaab et al. (2024) examined 48 studies focusing on ML-based detection of anxiety, depression, and stress, particularly in undergraduate students. While models showed high internal accuracy (over 70%, with anxiety-specific performance ranging from 53.7 to 98%), a critical limitation was the near-total absence of external validation, leading to a “very low” certainty of evidence. However, Ancillon et al. (2022) specifically reviewed anxiety detection using physiological biosignals like EEG, ECG, and electrodermal activity (EDA). Their findings highlighted that multimodal approaches integrating multiple signals, especially EDA and heart rate, generally achieved superior accuracies (between 55 and 98%) compared to univariate models. However, this review also noted persistent limitations such as small datasets, lack of benchmarking, and inconsistent preprocessing protocols hindering generalization. Razavi et al. (2023) conducted a broader scoping review of 98 studies on stress and related disorders, reinforcing observations of methodological convergence on supervised learning, limited personalization, and a dearth of interpretable models. They also identified issues like scarce real-world deployment and missing transparency metrics as common. These related works collectively converge on several recurring themes. First, is that there is limited external validation despite high internal accuracy. Seocnd, is the effectiveness of biosignal fusion, primarily confined to controlled lab settings. Third, is the methodological plateau in model architectures, with underprioritized interpretability, fairness, and contextual modeling. And lastly, there is a lack of standardized feature engineering, preprocessing, and benchmarking frameworks. While these studies effectively delineate existing problems, the current systematic review offers a uniquely compelling direction for future research by highlighting a notable scarcity in studies predicting anxiety before symptom manifestation. This is a crucial distinction, as most related works, like the primary studies they analyze, primarily focus on the classification or detection of existing anxiety. By emphasizing the need for a “paradigm shift from reactive classification to proactive risk forecasting,” this review's findings are more interesting and impactful for future research. It explicitly paves the way for studies aimed at early intervention and prevention, which could “significantly improve mental health outcomes and decrease healthcare costs.” This ambition for genuinely preventative solutions, akin to reliably “predict[ing] a storm before the clouds even gather on the horizon,” sets the current review apart from those primarily summarizing current detection capabilities. It provides a foundational roadmap, advocating for a shift toward anticipating anxiety onset, diversifying study populations, and integrating explainable AI for robust and ethical mental health support.

### Research challenges

A primary concern is the frequent reliance on relatively small and homogeneous datasets. Many models are trained on samples primarily composed of university students (Bhatnagar et al., 2023; Priya et al., 2020; Daza et al., 2023; Vitória et al., 2024; Morr et al., 2024), or older adults drawn from narrowly defined geographic regions (Benito et al., 2024; Basterfield and Newman, 2025). Such population biases significantly limit the generalizability of findings, raising questions about the models' effectiveness when applied to broader, more diverse populations.

Furthermore, the reporting of evaluation metrics across these studies is often inconsistent and, at times, inadequate. Several investigations rely solely on accuracy to assess model performance, despite the common presence of imbalanced class distributions in anxiety prediction tasks. More nuanced metrics such as the F1-score, Area Under the Curve (AUC), and recall provide richer insights into model behavior but are underutilized. Feature selection approaches also vary considerably; while some studies employ formal and well-established techniques like minimum Redundancy Maximum Relevance (mRMR) or information gain (Daza et al., 2023; Ku and Min, 2024), others either neglect this critical step or do not clearly describe their selection processes. This inconsistency in methodology further complicates replication and the assessment of comparative model quality.

Finally, the limited application of interpretability tools, such as SHapley Additive exPlanations (SHAP)–in the reviewed literature significantly constrains the clinical relevance of many machine learning models. Explainability is especially vital in mental health contexts where practitioners must understand and trust algorithmic predictions, particularly for early screening or real-time monitoring applications. The absence of transparent, interpretable frameworks undermines clinicians' confidence and reduces the likelihood of these models being integrated into practice. Taken together, these issues provide the need for standardized methodological guidelines, more rigorous validation on diverse cohorts, and the development of explainable models to advance machine learning's role in anxiety research meaningfully.

### Future research

The systematic review's findings offer a uniquely compelling vision for future research in anxiety prediction, distinguishing itself from related studies that primarily focus on existing detection or classification challenges. While other comprehensive analyses, such as those by Schaab et al. (2024), Ancillon et al. (2022), and Razavi et al. (2023), meticulously highlight methodological pitfalls like small datasets, lack of external validation, and interpretability issues, this review makes a critical conceptual leap. It underscores a “notable scarcity” in studies predicting anxiety before symptom manifestation, identifying this as a direction with “substantial potential for preventive healthcare.” This “paradigm shift from reactive classification to proactive risk forecasting” is profoundly more interesting and impactful for future endeavors. By systematically synthesizing current limitations and explicitly foregrounding the urgent need to predict anxiety onset to enable early intervention and reduce healthcare costs, this review provides a foundational roadmap. It clarifies how to move beyond simply identifying present anxiety toward genuinely preventative solutions, making its results exceptionally valuable for guiding the next generation of anxiety research. The suggested emphasis on diverse populations and explainable AI further ensures a robust and ethical future for ML in mental health.

## Conclusion and future scope

This study reviewed current applications of machine learning (ML) models for the prediction and classification of anxiety. Empirical findings across the literature indicate that ensemble-based approaches such as Random Forest and Gradient Boosting, along with Support Vector Machines (SVM) and model-stacking frameworks, offer high performance in detecting anxiety-related states. These models demonstrate strong classification capabilities, particularly when applied to multimodal and high-dimensional psychological datasets.

However, a notable limitation in the existing body of work is its predominant focus on classification–that is, distinguishing between anxious and non-anxious individuals based on already-present symptoms–rather than on early prediction or symptom trajectory modeling. Very few studies attempt to estimate the probability of anxiety onset in the future, a direction that holds substantial potential for preventive healthcare.

Future research should thus prioritize predictive modeling that aims to identify individuals at risk before anxiety symptoms become clinically manifest. This paradigm shift from reactive classification to proactive risk forecasting could enable timely interventions, reduce the burden on mental health infrastructure, and improve individual wellbeing. Additionally, there is a critical need to diversify study populations, incorporating a broader range of demographic variables such as age, region, socioeconomic background, and occupational context. This would facilitate the development of culturally sensitive and contextually relevant ML models, increasing their applicability and fairness across populations.

In summary, the integration of explainable, generalizable, and ethically aligned ML systems for anticipatory anxiety prediction represents a promising yet underexplored avenue for future interdisciplinary research.

## Data Availability

The data analyzed in this study is subject to the following licenses/restrictions. The systematic review included the conclusions drawn in the paper without analysis the original datasets. Access to the datasets may be restricted by the original authors or hosting institutions and may require permission for use. Requests to access the data should be directed to the corresponding authors or the institutional repositories listed in the respective publications: Bhatnagar et al. (2023) https://www.scribd.com/document/691271255/RG-Bhatnagar-et-al-2023-Learning-to-live-with-an-unruly-consuming-body-JCR; Priya et al. (2020) https://www.ncbi.nlm.nih.gov/pmc/articles/PMC7474906/; Daza et al. (2023) Email: ericjdaza@statsof1.org, https://www.medrxiv.org/content/10.1101/2023.03.13.23287212v1; Sau and Bhakta (2017) Email: arkaprabhasau@gmail.com, https://ietresearch.onlinelibrary.wiley.com/doi/full/10.1049/htl.2016.0096; Mohamed et al. (2023) https://journals.sagepub.com/doi/full/10.1177/23779608231189944; Qasrawi et al. (2022) https://www.bmcurol.biomedcentral.com/articles/10.1186/s12894-022-01133-1; Basterfield and Newman (2025) https://www.midus.wisc.edu/wp-content/uploads/2024/04/2976.pdf; Ku and Min (2024); Benito et al. (2024) https://www.psych.uni-goettingen.de/de/translational/publikationen/mechanisms-of-change-in-exposure-therapy-for-anxiety-and-related-disorders-a-research-agenda/%40%40download/pdf_file/benito-et-al-2024-mechanisms-of-change-in-exposure-therapy-for-anxiety-and-related-disorders-a-research-agenda.pdf; Fukazawa et al. (2019) https://www.sciencedirect.com/science/article/pii/S1532046419300693; Portugal et al. (2019) https://www.mdpi.com/2227-9032/11/5/659; Bailey and Strunk (2023) Email: strunk.20@osu.edu, https://www.onlinelibrary.wiley.com/doi/full/10.1002/jclp.23555; and Perpetuini et al. (2021) https://www.peerj.com/articles/10448/.
