# Supplementary material for: Machine learning approaches to anxiety detection: trends, model evaluation, and future directions
Source: Front Artif Intell. 2025 Oct 21;8:1630047. doi: 10.3389/frai.2025.1630047 (PMC12582962; doi:10.3389/frai.2025.1630047)
Supplement: Supplementary file 1 [file Supplementary_file_1.zip › Supplementary material/Supplementary material.pdf]

Supplementary Material

Figure 1 outlines the top-ranking countries, showcasing the USA and India leading in publications, with China also holding a prominent position. Moreover, Table 2 further highlights that the United States has many very productive institutions in this area, while institutions from Bangladesh and Saudi Arabia are achieving noteworthy results, too.

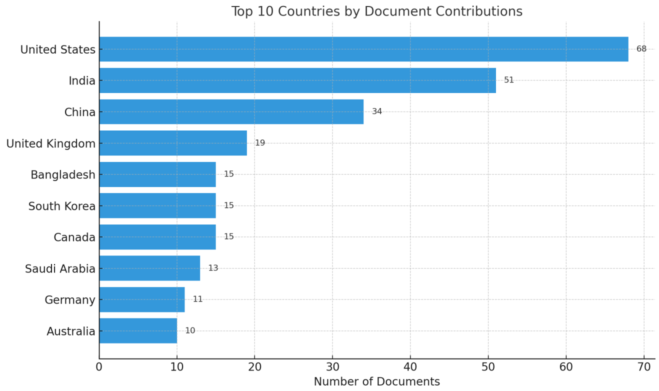

Figure 1. Documents by country

Google Scholar is a web search engine that was launched in 2004. It indexes scholarly literature in various publishing formats and across various academic disciplines. According to a recent study by Gusenbauer [2019], Google Scholar contains approximately 389 million documents, including articles, citations, and patents, making it one of the world's largest academic search engines. Google Scholar provides access to content from a variety of sources, including both freely accessible material and content that may require subscriptions, such as Scopus. It is a valuable tool for researchers and academics to discover scholarly publications and citations.

Table 1. Scopus dataset properties

Papers	255
Authors	161
Subject Area	24
Institutions	215
Source	4
Countries	57

Table 2. Top institutions by the number of publications in Scopus database

Rank	Institution	Total publications	Country
1	University of Pittsburgh	7	US
2	University of Pittsburgh School of Medicine	6	US
3	Daffodil International University	6	BD
4	Princess Nourah Bint Abdulrahman University	6	SA
5	Inje University	5	KR
6	University of Toronto	5	CA
7	University of Melbourne	5	AU
8	UConn Health	4	US
9	University of Washington	4	US
10	University College London	4	UK

Additionally, Table 3 identifies the Journal of Affective Disorders as the leading journal in 2021-2022, primarily focusing on medicine, except for Scientific Reports, a multidisciplinary journal. Moreover, Table 4 elucidates the frequency of combined keywords, emphasizing the prevalence of 'Forecasting' in articles related to 'Machine learning' and 'Learning systems,' despite certain keywords like 'anxiety' not being represented.

Table 3. The top 10 most productive journals by the number of publications

Rank	Journal	Publ.	Citations	CS 2022	Publ. on the topic
1	Journal of Affective Disorders	4862	46,714	9.6	11
2	Psychological Medicine	1213	16,437	13.6	5
3	Plos One	63,236	377,961	6.0	5
4	JMIR Formative Research	1541	3186	2.1	4
5	Frontiers in Public Health	8534	32,772	3.8	3
6	Frontiers in Psychiatry	7762	41,540	5.4	3
7	BMC Psychiatry	2406	13,106	5.4	3
8	Translational Psychiatry	1841	18,827	10.2	2
9	Smart Health	171	836	4.9	2
10	Scientific Reports	86,190	648,244	7.5	2

Table 4. Keyword Occurrence

Rank	Keyword	Occurrence
1	Machine learning	167
2	Depression	139
3	Human	98
4	Article	72
5	Female	65
6	Forecasting	61
7	Adult	60
8	Male	59
9	Mental Health	49
10	Learning systems	49

Table 5. Keywords Co-occurrence

	Machine learning	Depression	Human	Article	Female	Forecasting	Adult	Male	Mental Health	Learning systems
Machine learning	-	108	82	65	58	41	51	46	36	27
Depression	108	-	76	53	51	29	47	45	26	24
Human	82	76	-	71	64	9	60	58	17	7
Article	58	53	71	-	55	4	49	50	11	4
Female	52	51	64	55	-	3	49	59	11	4
Forecasting	41	29	9	4	3	-	3	3	13	25
Adult	51	47	60	49	55	3	-	45	9	3
Male	46	45	58	50	59	3	45	-	11	4
Mental Health	36	26	17	11	11	13	9	11	-	11
Learning systems	27	24	7	4	4	25	3	4	11	-

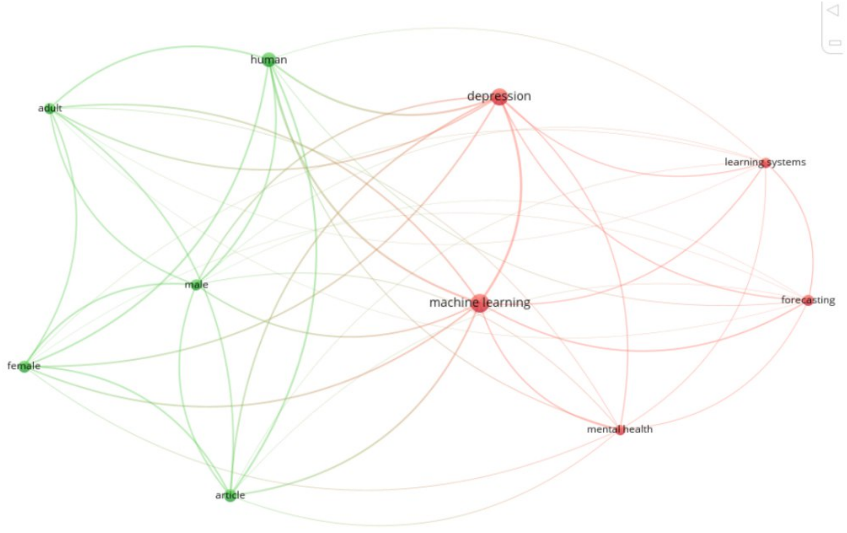

Figure 2. Keywords co-occurrence

Scopus allows us to determine the top 10 most productive authors. For instance, Table 6 presents a list of authors who have contributed the most articles on the subject of Anxiety prediction with ML. Notably, no clear leader has a significantly higher number of articles than the rest. The top authors have written between 2 to 4 articles, which is a relatively small number of publications. Clearly, most of these top authors, specifically 7 out of 10, are affiliated with institutions in the United States.

Table 6. The top 10 prolific authors in anxiety prediction with the ML research area

Number	Author	Publ.	TP	h-index	Current Affiliation	Country
1	Byeon, H.	4	118	16	Inje University, Gimhae	KR
2	Ware, S.	3	7	4	University of Richmond	US
3	Wang, B.	3	109	32	University of Connecticut, Storrs	US
4	Russell, A.	3	159	26	University of Connecticut, Storrs	US
5	Morillo, R.	3	9	5	University of Connecticut, Storrs	US
6	Kamath, J.	3	35	13	UConn Health, Farmington	US
7	Bi, J.	3	135	31	University of Connecticut, Storrs	US
8	van Noort, B.	2	35	14	MSB Medical School Berlin, Berlin	DE
9	van Hemert, A.M.	2	225	51	Leids Universitair Medisch Centrum	NL
10	Yue, C.	2	15	9	University of Connecticut, Calhoun	US

```
# Assume you have some base algorithms, a combiner algorithm, and datasets
base_models = [logistic_regression, decision_tree_classification, ...]  # for classification

stacking_train_data = matrix(row_length=len(target), column_length=len(base_models))
stacking_test_data = matrix(row_length=len(test), column_length=len(base_models))

# Loop through base algorithms and populate stacking datasets
for i, base_model in enumerate(base_models):
    stacking_train_data[:, i] = base_model.fit(train_data, target).predict(train_data)
    stacking_test_data[:, i] = base_model.predict(test_data)

# Use the combiner algorithm to make final predictions
final_predictions = combiner_model.fit(stacking_train_data, target).predict(stacking_test_data)
```

Figure 3. Pseudocode of a stacked generalization

REFERENCES

Gusenbauer, M. (2019). Google scholar to overshadow them all? comparing the sizes of 12 academic search engines and bibliographic databases. *Scientometrics*, 118:177–214.
